# Effect of a Multifaceted Intervention on Children’s Body Image and the Mediating Role of Body Image in Changes in Adiposity Indicators

**DOI:** 10.3390/nu15183951

**Published:** 2023-09-12

**Authors:** Jinlang Lyu, Zhongshang Wan, Zheng Liu, Shuang Zhou, Xiangxian Feng, Aiyu Gao, Yi Lin, Fang Zhang, Haijun Wang

**Affiliations:** 1 Department of Maternal and Child Health, School of Public Health, Peking University, National Health Commission Key Laboratory of Reproductive Health, Beijing 046000, China; jinlanglyu@bjmu.edu.cn (J.L.); 18101357806@163.com (Z.W.); liuzheng@bjmu.edu.cn (Z.L.); zhoushuang0601@bjmu.edu.cn (S.Z.); 2 Peking University Health Science Center-Weifang Joint Research Center for Maternal and Child Health, Weifang 261000, China; 3 Changzhi Medical College, Changzhi 046000, China; xfeng66@163.com; 4 Dongcheng Primary and Secondary School Health Care Center, Beijing 236499, China; qmx518@163.com; 5 Urumqi Primary and Secondary School Health Care Center, Urumqi 830003, China; 6 Mentougou Primary and Secondary School Health Care Center, Beijing 102300, China; zhf-1250@126.com

**Keywords:** children, obesity, intervention, body image, mediating effect

## Abstract

Besides genetic factors and energy-related behaviors, psycho-cultural factors are also important in obesity etiology. Previous studies have suggested that improving body image might be an effective method for managing body weight. Thus, this study aimed to evaluate the effects of a multifaceted intervention on the body image of children and explore whether body image played a mediating role in changes in adiposity indicators. This study was embedded in a cluster randomized controlled trial, involving 1287 children from 24 primary schools in three cities in China (Beijing, Changzhi and Urumqi). The 9-month multifaceted intervention on childhood obesity included five components (three targeted children and two targeted environments), and randomization was performed by an independent person who was blinded to the schools. Two indicators (body size perception and body size expectation) were chosen to characterize body image and were measured by Ma figural stimuli at baseline and the end of the trial. Changes in body image indicators were classified as conducive to weight loss or not. Other anthropometric measures and self-reported behaviors were also collected at both time points. Generalized linear mixed models were used in the analyses. Compared to the controls (*n* = 648), the proportion of body size perception conducive to weight loss increased in the intervention group (*n* = 639), with an odds ratio of 2.42 (95%CI: 1.70~3.45, *p* < 0.001). The proportion of body size expectation conducive to weight loss also increased more in the intervention group than in the controls (OR = 1.74, 95%CI: 1.14~2.66, *p* = 0.010). In children whose baseline nutritional status was “normal weight with higher BMI” or “overweight/obese”, the improvements in body size perception and body size expectation partly mediated the association between the intervention and changes in BMI, BMI Z score, waist circumference and body fat percentage (*p* < 0.05). This multifaceted study effectively improved the body image of children, which, in turn, led to beneficial changes in adiposity indicators. For the first time, body size perception and body size expectation have been confirmed to be important factors associated with the beneficial effect of a childhood obesity intervention, suggesting that body image components should be generalized in the future.

## 1. Introduction

Childhood obesity is a global health problem. The number of obese children and adolescents around the world has increased ten-fold in the past four decades from 1975 to 2016 [1]. In China, the prevalence rate of overweight and obesity among school-age children increased from 5.3% in 1995 to 20.5% in 2014 [2]. Childhood obesity might affect children’s physical and psychological health, academic attainment, quality of life, and can also increase the risk of metabolic diseases and cancer in adulthood [3,4,5,6]. There is an urgent need to develop effective intervention strategies for childhood obesity.

Besides genetic factors and energy-related behaviors (like diet, sedentary time and physical exercise), psycho-cultural factors are also important in obesity etiology [7,8]. Researchers have pointed out that body image is a psycho-cultural factor and might serve as an important motivation for strategies of weight modification [9]. Body image is a multifaceted psychological construct involving self-perception and self-attitudes on the physical aspects of the body [10]. These include thoughts, beliefs, feelings and behaviors. As experiences of body image could have far-reaching effects on human development, the international literature has revealed a growing research interest in body image over the past decade. Body image distortions were found to be common in children and adolescents (especially in those with abnormal weight statuses) [11,12].

Previous studies have shown that the negative subjective evaluations or experiences of one’s physical appearance may increase the risk of unhealthy behaviors, such as a sedentary lifestyle and eating disorder [11], and influence weight-loss attempts or weight-management behaviors [13]. Cross-sectional evidence has revealed that body image, such as the underestimation of body size, was related to less weight control behaviors, higher body mass index (BMI) or weight status [9,14]. Researchers also found that children who expected their body size to become fatter or were satisfied with their body size had less weight loss behaviors than those dissatisfied and expecting to be thinner, as the self-desired discrepancies gave them motivation [15,16]. Thus, different body images, for children, might exert two different impacts on their behaviors: conducive to weight loss (that is, more likely to result in efforts to lose or manage weight) or not. A few longitudinal studies have investigated the impact of body image on childhood obesity, which have had inconsistent results and have focused mainly on BMI without considering other physical indicators [12,17,18]. In intervention studies, only one study has investigated the relationship between changes in body size expectation and BMI Z scores in children aged 5~9 years old [19]. Very little is known about the impact of body image on obesity indicators in intervention studies. Previous studies have suggested that changes in body image might serve as a mediator and contribute to the improvement in obesity indicators in an intervention, but there is lack of evidence [20,21].

The Diet, Exercise and Cardiovascular Health—Children (DECIDE-Children) program was a cluster randomized clinical trial for a multifaceted intervention, including five components targeting three levels of children, school and family. A total of 1392 children from 24 primary schools across three socioeconomically distinct regions in China participated in the intervention. During the intervention, children were helped to establish a positive body image using health education activities, regular weight monitoring and receiving feedback. Our primary study showed that the DECIDE-Children program was effective in reducing BMI and the prevalence of obesity [22]. These effects are encouraging, and it is necessary to further investigate whether body image mediates the intervention effects.

Based on the data from DECIDE-Children program, this study mainly had two objectives. Firstly, we evaluated the effect of an intervention on the improvement in children’s body image. And then we analyzed the mediating role of the changes in body image between the intervention and the changes in adiposity indicators, in order to provide scientific evidence for incorporating body image in a multifaceted intervention in the future.

## 2. Materials and Methods

### 2.1. Study Design and Participants

This study was embedded within the DECIDE-Children study. Using the cluster random method and stratified by district (urban or suburban), 24 schools (8 schools in each region) were divided in a 1:1 ratio into the intervention group or control group, involving 1392 eligible children aged 8 to 10 years. A researcher at the Clinical Research Institute of Peking University, who was blinded to the schools, was in charge of the randomization process. The study was reviewed and approved by Peking University Institutional Review Board (IRB00001052–18021), and the informed consent of all children and parents was obtained before their participation in the study.

### 2.2. Description of Intervention

Based on the social ecological model, the multifaceted intervention for the prevention of obesity among school-aged children included five components (three targeting children and two targeting their school and family environment) and lasted for 9 months from 11 September 2018 to 30 June 2019. A smartphone app was used to strengthen parental involvement and cooperation. See the published research protocol for a detailed description of the intervention methods [23].

In terms of improving children’s body image, the intervention measures mainly included health-education activities, regularly monitoring children’s weight and giving personal feedback. Children’s health-education activities were carried out 10 times in total (once every 2~3 weeks, each lasting for 40 min), in which children were taught to establish the correct measurement and an evaluation of their own body shape. To improve their motivation on control of their weight gain, children and their parents were also educated about the adverse effects of obesity on health and life. Weight monitoring and feedback were carried out by school doctors or teachers. They measured the height and the weight of each child once a month, and gave personal feedback on actual weight status to children and their parents through a smartphone app regularly. Children were also told how to evaluate their nutritional status based on their BMI, and encouraged to measure their weight by themselves once a week. The control schools just took regular school health and physical education activities. In order to avoid unnecessary weight loss and help children form an appropriate attitude towards their body image, the intervention took targeted measures for children with different nutritional statuses. Children with overweight or obesity were guided to lose weight to achieve a healthier weight status and a thinner body size. Children of a normal weight with a BMI higher than the median were guided to understand the difference between their current weight and the cutoff values of overweight and obesity, keep their body size and actively control the rapid growth of their weight. Children of a normal weight with a BMI lower than the median, or those underweight, were taught to develop healthy eating and exercise habits, rather than control their weight or pursue excessive slimness.

### 2.3. Measurements

Two indicators (body size perception and body size expectation) were used to characterize two important dimensions of children’s body image: self-perceived weight status and body image dissatisfaction.

**Figure 1:**
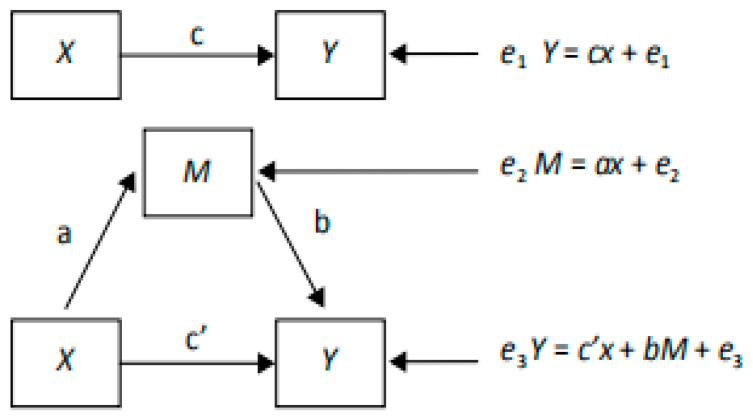
Model of the mediating effect pathway.

Body size perception was measured using Ma figural stimuli, which were developed based on Collins figural stimuli [24], and adapted to the appearance characteristics and habits of Chinese people (See Figure S1) [25]. We selected the arrays of pictures designed for children. There were two arrays of pictures for boys and girls, respectively. Each array contained seven black-and-white line figures ranging from very thin to very fat, which were numbered from 1 to 7. Children were asked to select one picture that fit best with the following statements: “Which figure do you think looks most like your current body size?”. If the children perceived themselves to be consistent with their real nutritional status (children with normal weight chose Figures 3~5; children with overweight chose Figure 6; and children with obesity chose Figure 7), their body size was perceived correctly. If the children perceived themselves to be fatter than their real nutritional status (children with normal weight chose Figures 6~7; children with overweight chose Figure 7), their body size was overestimated. If the children perceived themselves to be thinner than their real nutritional status (children with normal weight chose Figures 1~2; children with overweight chose Figures 1~5; and children with obesity chose Figures 1~6), their body size was underestimated. Body size expectation was also evaluated based on the Ma figural stimuli [25]. The children were asked “Which figure do you want to look like (ideal body image)?” and then chose the corresponding number of the figure. Their body size expectation score was obtained by subtracting the figure number of their ideal size from the number for their current body size that children chose. If the result was zero, children were thought to be satisfied with their body image. If the score was <0 or >0, children were thought to be dissatisfied with their body image. In this study, the score ≥ 2 represented “moderate dissatisfaction and expectation to be thinner”.

According to its association with weight-control behaviors, these two body image indicators were coded as binary variables (conducive to weight loss or not). The underestimation of body size was coded as 0 (not conducive to weight loss). The overestimation and correct estimation of body size were coded as 1 (conducive to weight loss). Moderate dissatisfaction and expectation to be thinner was coded as 1 (conducive to weight loss). Changes in these two indicators were also coded as binary variables. The changes conducive to weight loss were coded as 1 (including two cases: (1) the body image indicator was conducive to weight loss at both baseline and the final survey; (2) the body image indicator was not conducive to weight loss at baseline, but conducive to weight loss at the end of the trial). The remaining conditions were considered as changes that were not conducive to weight loss and coded as 0.

The children’s weights and heights were measured to the nearest 0.1 kg and nearest 0.1 cm. Their waist circumferences were measured to the nearest 0.1 cm with Myotape body measure tape. Their body fat percentages were measured to the nearest 0.1% with a body component instrument (Tanita MC-780 MA).

Body mass index (BMI) was calculated by dividing the weight (kg) by the square of the height (m^2^). The BMI Z score was calculated according to the World Health Organization (WHO) standards [26]. According to Chinese references and standards of overweight, obesity and underweight [27,28], the nutritional statuses of the children was classified as underweight, normal weight, overweight or obesity using age- and sex-specific BMI percentiles. Among normal-weight children, if their current BMI ≥ the median BMI of children with the same sex and age, their nutritional status was classified as “normal weight with BMI higher than the median”, and if their BMI < the median, their nutritional status was classified as “normal weight with BMI lower than the median” [29].

### 2.4. Statistical Analyses

Statistical analyses were conducted using the R 4.1.0 software. Categorical variables were described by the number and percentage of cases. Continuous variables with normal distribution were described by the mean and standard deviation. General characteristics were compared using a *t*-test for continuous variables, with Chi-Square tests for categorical variables.

The effect of the intervention on body image was analyzed using a generalized linear mixed model, taking the intervention or control group as the independent variable and the children’s body size perception and expectation at the end of the trial as the dependent variable, respectively. The children’s age, gender, nutritional status and body image indicators at baseline were adjusted in the model, along with a school-level random intercept considering the correlation due to the clustering of children within schools.

The mediating effect of the change in the children’s body image between the multifaceted intervention and changes in adiposity indicators was analyzed using the mediation method. As shown in Figure 1, the regression coefficient of the effect of factor X on dependent variable Y was determined in Path c (total effect), and the coefficient of the effect of factor X on dependent variable Y when adjusting for factor M was determined in Path c’ (direct effect). The product of coefficients in pathway a and b (a×b) represented the indirect effect. In this study, the change in the children’s body size perceptions or body size expectations was considered as a mediator factor (M), the intervention or control group assignment was considered as an independent variable (X), and the changes in adiposity indicators were considered as dependent variables (Y). Subgroup analysis was conducted according to their nutritional status at baseline (normal weight with BMI lower than the median, normal weight, with BMI higher than the median, overweight or obese).

## 3. Results

### 3.1. General Characteristics

As Table 1 showed, there were no significant differences in gender, age, BMI, or other characteristics between the intervention group and the control group at baseline (all *p* values > 0.05).

### 3.2. The Effect of Intervention on the Children’s Body Image

As shown in Table 2, after a 9-month intervention, the proportion of the children’s body size perception conducive to weight loss increased by 16.2% in the intervention group (from 58.0% to 74.2%) while the proportion in the control group only increased by 6.5% (from 52.3% to 58.8%), with an odds ratio of 2.42 (95%CI: 1.70, 3.45, *p* < 0.001). Compared with the control group, the children’s body size expectation conducive to weight loss also improved more in the intervention group (OR = 1.74; 95%CI: 1.14~2.66; *p* = 0.010).

### 3.3. Mediating Role of Body Image between the Intervention and the Changes in Adiposity Indicators

Among the children with overweight or obesity at baseline, it was found that the changes in the children’s body size perception played a partial mediating role in the association between the intervention and the changes in BMI, BMI Z score and body fat percentage. And the indirect effects were −0.08 (95%CI: −0.15~−0.03, *p* = 0.002), −0.04 (95%CI: −0.06~−0.02, *p* < 0.001) and −0.28 (95%CI: −0.50~−0.10, *p* < 0.001), respectively (see Table 3). Among the children of “normal weight with higher BMI” at baseline, it was also found that the changes in the children’s body size perception played a partial mediating role in the improvement in the four adiposity indicators. Compared with the “normal weight with lower BMI” group, the indirect effect of the change the children’s body size perception in the “normal weight with higher BMI” group and “overweight/obese” group were similar. Therefore, these two groups were combined to calculate the proportion of the mediating effect. Among these children, the changes in body size perception mediated 14.81% (TE: −0.54, IE:−0.08, *p* < 0.001), 17.65% (TE: −0.17, IE: −0.03, *p* < 0.001), 9.30% (TE: −1.72, IE: −0.16, *p* < 0.05), and 18.03% (TE: −1.22, IE: −0.22, *p* < 0.01) of the association between the intervention and changes in the BMI, BMI Z score, waist circumference and body fat percentage, respectively.

The mediating effect of body size expectation was also found in children with overweight or obesity. The indirect effects for the BMI, BMI Z score and body fat percentage were −0.04 (95%CI: −0.08~−0.01, *p* = 0.032), −0.02 (95%CI: −0.04~−0.01, *p* = 0.003) and −0.15 (95%CI: −0.30~−0.03, *p* = 0.006), respectively (see Table 3). Among children in the “normal weight with higher BMI” group, although not significant, the direction of the mediating effect was similar with the “overweight/obese” group. When combining these two groups together, the change in body size expectation mediated 7.41% (TE: −0.54, IE: −0.04, *p* < 0.01), 11.76% (TE: −0.17, IE: −0.02, *p* < 0.01), 5.81% (TE: −1.72, IE: −0.10, *p* < 0.05), and 7.26% (TE: −1.24, IE: −0.09, *p* < 0.05) of the association between the intervention and the changes in the BMI, BMI Z score, waist circumference and body fat percentage, respectively.

## 4. Discussion

After the 9-month intervention, the proportions of the children’s body size perceptions and body size expectations conducive to weight loss in the intervention group increased more than that in the control group. The multifaceted intervention could effectively improve the children’s body image. In the children whose baseline nutritional status was “normal weight with higher BMI” or “overweight/obese”, the changes in body image conducive to weight lose partly mediated the improvement by the intervention on the children’s adiposity indicators. That is, this multifaceted intervention for childhood obesity can partially decrease the level of children’s adiposity indicators through improving their recognition of and attitude towards their own body size.

Current obesity studies have rarely found a positive effect of interventions on children’s body image [30,31,32,33]. In addition to differences brought by populations and regions, it may also be because of a lack of intervention measures aimed at improving children’s body image. In the studies mentioned above, the main intervention goal was weight loss and the intervention measures included enhancing physical activity, selecting healthy food, and reducing sedentary behavior, without a specific component to teach individuals to form an accurate perception of their body size. In the DECIDE-Children program, guided by social ecological theory, our research group designed a number of intervention measures to help children to perceive and treat their body size correctly: delivering education materials and lessons on evaluating nutritional status, weekly training for children to monitor their own weight, giving monthly feedback on weight status and behaviors, etc. Systematic review evidence have supported the finding that multiple session interventions for children were more effective than a single session on the promotion of body image, and that school-based psychoeducational methods could have positive effects as well [34]. With these repeated and regular training sessions for children and their parents, they may be more likely to transform correct recognition into healthy habits and perform better in weight management.

To our knowledge, this is the first study to identify the mediating role of body image in the effects of an intervention on childhood obesity. Possible explanations could be found from health behavior theories such as the Transtheoretical Model and Health Belief Model. According to the Transtheoretical Model [35], guiding children to evaluate their weight status and understand the harm of obesity will help them to make the step from the stage of Pre-contemplation to the stage of Contemplation (that is, develop a desire to control their weight and form healthy habits). In addition, researchers have pointed out that components in the Health Belief Model, such as susceptibility and barriers, could be addressed through healthy weight education that included topics of body image and instruction in time management for planning healthy behaviors into a daily schedule [36]. Along with a better school environment and parents’ help, it was easier for them to improve self-efficacy and take action to lose weight. Previous studies have also found that correct body size perception was related to more active weight-management attempts [37].

The study results revealed that an improvement in the children’s body image was associated with changes in adiposity indicators. Body image conducive to weight loss led to larger decreases in their BMIs, BMI Z scores, waist circumferences and body fat percentages. In addition to obesity, the association between body image indicators and mental health was also found in previous studies. Body dissatisfaction was associated with quality of life impairment and shown to impact emotional well-being [38]. Positive changes in body image not only reduced the risk of obesity-related health outcomes, but also exhibited a greater influence on mental health among individuals with obesity than other potential variables [39]. This reminded us that this has become a public health concern and should be incorporated in future interventions to fully improve the physical and mental health of children and adolescents. However, most of the current research has supported weight loss as the main goal and ignored children’s improvement in body image indicators. Collaboration with experts from clinical psychology and adding related content in health course curriculums for stakeholders (i.e., teachers, school doctors, parents and researchers) might be effective ways to improve the awareness and understanding of the importance of body image in obesity prevention among obesity researchers [39,40].

Given the increasingly high prevalence and adverse impact of mental health disorders among children with overweight and obesity, the overall goal of obesity treatment in this vulnerable population is to prevent negative lifelong health complications, both medical and psychological. This suggests that psychological health should be considered an important intervention outcome, equal to physiological health. Assessing emotional well-being is also warranted in obesity interventions to further confirm the benefits in multiple dimensions of children’s overall health brought by integrated interventions specifically targeting body image. Negative body image is thought to be a shared risk factor of obesity and disordered-eating behavior [41]. Researchers have also identified that mental health problems often co-exist with obesity and have shared pathways of relevance to obesity [40]. This commonality provides chances for integrated health-promotion programs to reach the victims of negative body image, disordered-eating behavior and adiposity simultaneously. Intervention by modifying shared risk factors is an expeditious and efficacious method and has the potential to successfully impact multiple health outcomes [41]. Approaches including conducting cognitive behavioral therapy, improving family engagement and more frequent contact with study personal showed great feasibility and potential [42].

Generally speaking, when children recognized that they were overweight or expected to be thinner, they would be more likely to exhibit weight control behaviors, such as a healthy diet and physical exercise [13,43,44]. However, contrary to common sense, in some prospective studies researchers tended to find out that children who recognized they were overweight had more weight gain and a higher incidence of obesity in the future [45,46,47]. A possible explanation was that these children chose unhealthy and extreme weight-control behaviors like skipping meals without guidance and binge eating, resulting in subsequent weight gain and psychosocial problems [46,47]. Another important reason was that there was still a gap between the transition from consciousness to behavior among the children [44,48]. Children’s self-control ability is relatively weak and changes in their lifestyle patterns require more willpower than adults when facing such an “obese environment” in China [49,50,51,52]. The DECIDE-children intervention program also gave supervision and detailed instructions on the formation of healthy lifestyle patterns afterwards. Thus, these results reminded us that changing children’s body image is only an important first step: guidance afterwards on the internalization of body image through healthy weight management and lifestyle improvements is also an important.

The main strength of this study included the study design being based on a cluster randomized controlled trial. The intervention longitudinal data not only allowed comparisons between baseline and the end of the trial, but also between the intervention group and the control group, which could provide more scientific and persuasive evidence on sequential associations. In addition, a generalized linear mixed model was used and the correlation due to the clustering of children within schools was considered in the model. Finally, our study conducted mediation analyses and explored the mediating role of body image between the intervention and the changes in adiposity indicators. For the first time, changes in body size perception and body size expectation were confirmed to be important factors associated with the beneficial effects of an intervention.

This study had some limitations. Firstly, as the age range of the children was narrow (8~10 years old), the effects in children of other age groups needs to be further explored. Secondly, the intervention duration was relatively short (9 months), which limited the opportunity for analysis of the long-term impact of the intervention on the children’s body image, and to explore the association between the children’s body image and childhood obesity on a larger time scale. However, a nine-month intervention does not belong to the short-term intervention study category (less than three months). Thus, the findings from this study still have significance for future research. Additionally, disordered-eating behavior which was found to be closely associated with body image was not considered in this study. In addition to adiposity indicators, exploring the influence of body image on these psychosocial outcomes in the future is also needed to promote overall health of children.

## 5. Conclusions

This multifaceted intervention for childhood obesity increased the extent of the children’s body image that was conducive to weight loss. The changes in body image played a mediating role between the intervention and the children’s adiposity indicators. These results help to clarify the mechanisms of an effective intervention on childhood obesity and provide scientific evidence for incorporating body image in a multifaceted intervention in the future.

## Figures and Tables

**Table 1:** General characteristics of children at baseline.

	Intervention (*n* = 648)	Control (*n* = 639)	*P*
Number of schools	12	12	-
Number of children/school, median(range)	54(47~60)	53(46~58)	-
Sex, *n* (%)			0.167
Male	329 (50.8)	350 (54.8)	
Female	319 (49.2)	289 (45.2)	
Region, *n* (%)			0.448
Beijing	234 (36.1)	210 (32.9)	
Changzhi of Shanxi	185 (28.5)	187 (29.3)	
Urumuqi of Xinjiang	229 (35.3)	242 (37.9)	
Nutritional status			0.232
Normal weight with lower BMI, *n* (%)	142 (21.9)	130 (20.3)	
Normal weight with higher BMI, *n* (%)	242 (37.4)	216 (33.8)	
Overweight and obesity *n* (%)	264 (40.7)	293 (45.9)	
Age (year)	9.6 (0.4)	9.6 (0.4)	0.748
BMI (kg/m^2^)	18.8 (3.6)	19.0 (3.6)	0.315
BMI Z score	0.8 (1.3)	0.9 (1.3)	0.244
Waist circumference (cm)	65.9 (10.0)	66.3 (10.9)	0.434
Body fat percentage (%)	21.4 (10.2)	21.8 (10.2)	0.542

**Table 2:** The effect of intervention on the children’s body image ^a^.

	Baseline	End of Trial	Δ	OR (95%CI)	*p*
Body size perception					
Control group	334 (52.3%)	376 (58.8%)	6.5%	Ref	
Intervention group	376 (58.0%)	481 (74.2%)	16.2%	2.42 (1.70, 3.45)	<0.001 ^*^
Body size expectation					
Control group	162 (25.4%)	125 (19.6%)	−5.8%	Ref	0.010 ^*^
Intervention group	159 (24.5%)	164 (25.3%)	0.8%	1.74 (1.14, 2.66)	

**^a^** Generalized linear mixed model was used, adjusting for the children’s body image, age, sex, and nutritional status at baseline, with the clustering effect of the school level. Δ: Change in the children’s body size perception or body size expectation (proportion conducive to weight loss) from baseline to end of trial. * *p* < 0.05.

**Table 3:** Mediating effect of the change in children’s body image between the intervention and changes in adiposity indicators ^a^.

	BMI (kg/m^2^)		BMI Z Score		WC (cm)		BF (%)	
	Β (95%CI)	*p*	β (95%CI)	*p*	β (95%CI)	*p* Value	β (95%CI)	*p*
Body size perception ^b^								
Overweight/obese								
Indirect effect	−0.08 (−0.15, −0.03)	0.002 *	−0.04 (−0.06, −0.02)	<0.001 *	−0.16 (−0.39, 0.01)	0.081	−0.28 (−0.50, −0.10)	<0.001 *
Direct effect	−0.69 (1.07, 0.30)	0.001 *	−0.17 (−0.27, −0.08)	<0.001 *	−1.89 (−3.63, −0.18)	0.028 *	−1.10 (−2.19, 0.03)	0.055
Total effect	−0.77 (−1.15, −0.39)	<0.001 *	−0.21 (−0.30, −0.11)	<0.001 *	−2.05 (−3.77, −0.35)	0.014 *	−1.38 (−2.47, −0.27)	0.016 *
Normal weight with higher BMI								
Indirect effect	−0.09 (−0.19, −0.01)	0.025 *	−0.04 (−0.07, −0.01)	0.023 *	−0.16 (−0.37, −0.01)	0.028 *	−0.18 (−0.41, −0.02)	0.020 *
Direct effect	−0.16 (−0.36, 0.05)	0.132	−0.08 (−0.18, 0.02)	0.119	−1.11 (−2.39, 0.21)	0.097	−0.80 (−1.66, 0.05)	0.070
Total effect	−0.25 (−0.47, −0.03)	0.023 *	−0.12 (−0.22, −0.01)	0.022 *	−1.81 (−3.30, −0.31)	0.018 *	−0.98 (−1.87, −0.12)	0.025 *
Normal weight with lower BMI								
Indirect effect	0.00 (−0.02, 0.02)	0.832	−0.00 (−0.01, 0.01)	0.930	0.05 (−0.05, 0.21)	0.417	−0.01 (−0.08, 0.04)	0.726
Direct effect	−0.17 (−0.34, 0.01)	0.074	−0.11 (−0.22, 0.01)	0.073	−1.35 (−2.59, −0.09)	0.037 *	−0.70 (−1.33, −0.07)	0.027 *
Total effect	−0.17 (−0.34, 0.02)	0.079	−0.11 (−0.22, 0.01)	0.074	−1.30 (−2.56, −0.04)	0.044 *	−0.71 (−1.34, −0.08)	0.026 *
Body size expectation ^b^								
Overweight/obese								
Indirect effect	−0.04 (−0.08, −0.01)	0.032 *	−0.02 (−0.04, −0.01)	0.003 *	−0.13 (−0.33, 0.01)	0.080	−0.15 (−0.30, −0.03)	0.006 *
Direct effect	−0.74 (−1.09, −0.34)	<0.001 *	−0.19 (−0.29, −0.10)	<0.001 *	−1.99 (−3.83, −0.16)	0.033 *	−1.26 (−2.33, −0.20)	0.023 *
Total effect	−0.78 (−1.12, −0.38)	<0.001 *	−0.21 (−0.31, −0.11)	<0.001 *	−2.12 (−3.96, −0.28)	0.022 *	−1.41 (−2.48, −0.35)	0.013 *
Normal weight with higher BMI								
Indirect effect	−0.01 (−0.06, 0.03)	0.544	−0.01 (−0.04, 0.02)	0.527	−0.04 (−0.20, 0.09)	0.521	−0.03 (−0.14, 0.07)	0.538
Direct effect	−0.23 (−0.44, −0.02)	0.035 *	−0.10 (−0.19, −0.01)	0.042 *	−1.16 (−2.43, 0.06)	0.067	−0.93 (−1.83, −0.06)	0.035 *
Total effect	−0.24 (−0.46, −0.03)	0.028 *	−0.11 (−0.21, −0.01)	0.034 *	−1.20 (−2.47, 0.04)	0.058	−0.96 (−1.86, −0.08)	0.030 *
Normal weight with lower BMI								
Indirect effect	0.00 (−0.02, 0.03)	0.824	0.00 (−0.01, 0.02)	0.830	0.00 (−0.12, 0.11)	0.931	0.00 (−0.07, 0.07)	0.942
Direct effect	−0.17 (−0.34, 0.01)	0.065	−0.11 (−0.22, 0.01)	0.074	−1.31 (−2.61, −0.02)	0.045 *	−0.67 (−1.28, −0.06)	0.029 *
Total effect	−0.17 (−0.34, 0.01)	0.070	−0.11 (−0.22, 0.01)	0.077	−1.31 (−2.63, −0.03)	0.046 *	−0.67 (−1.28, −0.06)	0.030 *

^a^ Generalized linear mixed model was used, adjusting for children’s age, sex and the clustering effect of the school level; ^b^ Change in children’s body size perception or body size expectation was a binary variable. * *p* < 0.05.

## Data Availability

Data described in the manuscript, codebook, and analytic code will not be made available because the Peking University Institutional Review Board have not consented to this. In order to access more information on data analyses, contact the corresponding author at whjun@pku.edu.cn.

## Supplementary Materials

The following supporting information can be downloaded at: https://www.mdpi.com/article/10.3390/nu15183951/s1, Figure S1: Ma figural stimuli.
