# Supplementary material for: Effect of a Multifaceted Intervention on Children’s Body Image and the Mediating Role of Body Image in Changes in Adiposity Indicators
Source: Nutrients. 2023 Sep 12;15(18):3951. doi: 10.3390/nu15183951 (PMC10534561; doi:10.3390/nu15183951)
Supplement: Supplementary file 1 [file nutrients-15-03951-s001.zip › nutrients-2564921-supplementary.pdf]

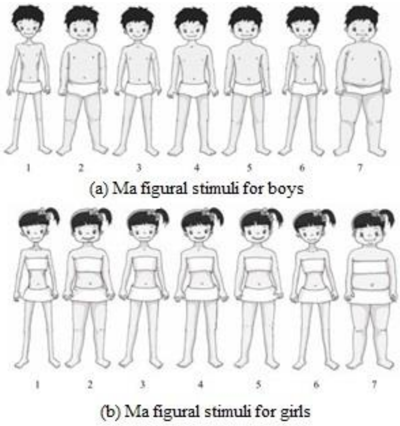

Supplementary Figure S1. Ma figural stimuli.
